# Dynamic Facial Health Predicts Psychological First Impressions with Applications to Tailored Treatments for Facial Paralysis

**DOI:** 10.3390/jpm15110530

**Published:** 2025-11-02

**Authors:** Nathaniel E. Helwig, Lauren N. Berry, Tessa A. Hadlock, Stephen J. Guy, Sofía Lyford-Pike

**Affiliations:** 1 Department of Psychology, University of Minnesota, Minneapolis, MN 55455, USA; 2 School of Statistics, University of Minnesota, Minneapolis, MN 55455, USA; 3 Department of Statistics, Grand Valley State University, Allendale, MI 49401, USA; berryln@gvsu.edu; 4 Department of Otolaryngology, Harvard Medical School, Boston, MA 02114, USA; 5 Department of Computer Science & Engineering, University of Minnesota, Minneapolis, MN 55455, USA; sjguy@umn.edu; 6 Department of Otolaryngology-Head and Neck Surgery, University of Minnesota, Minneapolis, MN 55455, USA

**Keywords:** face perception, interpersonal traits, rehabilitation, smile, social perception

## Abstract

**Background**: Past studies demonstrate that certain facial features systematically affect first impressions of psychological traits. However, no previous studies have examined how individual differences in facial health affect first impressions of psychological traits. **Methods**: In this study, we asked a large sample of fairgoers to give their first impressions of psychological traits in response to viewing videos of unilateral facial paralysis patients with varying degrees of facial functioning. Then, we used linear mixed-effects regression models to understand how individual differences in facial health predict first impressions. **Results**: Our results replicate previous findings regarding first impressions of faces, such as the attractiveness halo effect, as well as age (maturity) and gender (masculinity) effects. More importantly, our results reveal that facial health, as measured by a clinician-graded scale, is a significant predictor of first impressions. Specifically, we found that individuals with better dynamic facial health (as assessed by clinicians) were perceived to be more competent and more affiliative, but not more dominant, than individuals with lower levels of dynamic facial functioning. **Conclusions**: Our results have important implications for personalized medicine via the development and refinement of individually tailored therapies to improve facial reanimation surgery outcomes.

## 1. Introduction

### 1.1. Motivating Background

The questions of how and why facial features influence first impressions have sparked the interest of many [1,2,3,4,5,6]. Past studies have revealed robust findings regarding psychological impressions of facial characteristics. One well-established finding is the “attractiveness halo effect”, which is the finding that attractive people are perceived to have better psychological traits [7,8,9]. Specifically, people who are perceived to be more attractive are perceived to be more intelligent, sociable, and dominant—even though such perceptions may be inaccurate [8]—and such effects have been shown to exist across cultures [9]. These findings have motivated researchers to study the general and specific mechanisms that associate facial features with perceived psychological characteristics.

Theories of social perception offer general mechanisms by which people may associate facial features with psychological traits [10,11,12]. The ecological theory of social perception [13] suggests that the attractiveness halo effect is an example of overgeneralization. Specifically, the ecological approach predicts that people may overgeneralize judgements based on characteristics that are important for survival and/or functioning [14]. As an example, the ecological theory suggests that physical attractiveness may be an indicator of reproductive fitness, which is an important characteristic for species survival [1]. Such evolutionary adaptations can account for the overgeneralization of facial attractiveness to positive psychological traits and of facial anomalies to negative psychological traits [15].

### 1.2. Limitations of Past Work

The idea that people overgeneralize facial features important for survival and/or functioning can elucidate other well-known findings about first impressions [16]. For example, past research suggests that facial maturity is positively correlated with perceived competence [17]. Facial maturity may be an indicator of experience and wisdom, and perceived competence is important for electing leaders [18,19]. As another example, research suggests that facial masculinity is positively correlated with perceived dominance [20]. Facial masculinity may be an indicator of physical strength, and the quick identification of threatening faces is vital for survival [21].

Although past studies have provided general insights into how facial features affect first impressions, they have largely ignored specific details regarding how individual differences in facial function affect first impressions (e.g., [22,23,24]). This is due to two reasons. First, many past studies have focused on impressions drawn from photos of faces, which makes it impossible to examine the influence of dynamic aspects (e.g., [25,26]). There exist studies exploring how the medium of the presented information (e.g., photo vs. video) affects the first impressions of faces (e.g., [27,28,29,30,31]); however, this body of work does not explore how individual differences in facial health and function affect first impressions. Second, until recently, researchers have lacked a systematic scale for the quantification of individual differences in facial function. As a result, much of our current knowledge about psychological impressions of facial characteristics lacks information about the influence of facial health and function.

### 1.3. Present Research

We address this gap in the literature by exploring how individual differences in facial health relate to psychological first impressions. Specifically, the goals of this study are to determine (i) whether overall facial health is a relevant factor in forming first impressions of psychological traits and (ii) which aspects of facial health are most relevant for determining those first impressions. Given the well-known “attractiveness halo effect”, we explore the relationships between facial health and first impressions after statistically controlling for perceived attractiveness. We also control for age and gender effects, given that past research has demonstrated associations between first impressions and both facial maturity [17] and facial masculinity [20]. Controlling for covariates that are known to affect first impressions of faces allows us to explore how facial health affects first impressions above and beyond well-known effects.

We measure facial health using the eFACE scale [32], which is a clinician-graded measure of facial health and function. The eFACE scale grades facial health in terms of three subscores, Static, Dynamic, and Synkinesis, along with an overall facial health score. We hypothesize that, after controlling for the covariates of interest, (A) overall facial health will be significantly positively associated with the outcome variables, and (B) each subdomain of facial health will significantly contribute to first impressions, with the effect sizes ordered such that Dynamic > Static > Synkinesis. Note that hypothesis A is suggested by past research that demonstrates overall facial health is positively associated with perceived disfigurement [33,34]. Hypothesis B is proposed because the same past research has revealed that each subdomain of facial health affects first impressions of disfigurement, with resulting effect sizes in the aforementioned order.

We expect our results to be useful for advancing personalized medicine within the field of facial reanimation surgery and rehabilitation. In particular, our experimental design and analysis makes it possible to examine precisely how individual differences in facial health and function, as measured by the eFACE scale, influence first impressions of psychological traits. By studying the relative influence of each component of facial health, our approach makes it possible to directly study the influence of different facial function dimensions on psycho-social perceptions, which has the potential to enhance pre- and post-surgical outcomes. In particular, we anticipate our findings to be informative for facial reanimation surgeons and rehabilitative therapists, as they design facial movement exercises to help patients (re)learn to express psychologically and socially appropriate facial expressions.

## 2. Materials and Methods

### 2.1. Raters

Participants were fairgoers at the 2016 Minnesota State Fair who were recruited as they passed by the Driven to Discover building, a University of Minnesota facility on the fairgrounds. Participants were asked to report the number of alcoholic drinks they had consumed that day, so that inebriated fairgoers could be excluded from the analyses. We predetermined that those who reported consuming three or more drinks should be excluded from the analyses, which resulted in the exclusion of data from nine fairgoers. After removing these individuals, our sample consisted of 550 raters with the following demographics: 383 females (mean age, 40.01 years; range, 18 to 80), 164 males (mean age, 39.25 years; range, 18 to 86), and 3 who identified with the gender “other” (ages 18, 21, and 63 years). The three individuals who identified with a gender of “other” were excluded from the analyses, because three individuals is not enough to provide reliable effect estimates. Due to small amounts of missing data on the different response and/or predictor variables, our effective sample size differed slightly for each analysis: 538 raters for Competence, 533 raters for Affiliation, and 536 raters for Dominance.

### 2.2. Materials

#### 2.2.1. Patient Videos

Videos consisted of 61 patients with varying degrees of unilateral facial paralysis, ranging from low to high facial functionality. Each video lasted 3–5 s and was framed around the patient’s face and upper body with a blue backdrop. The videos showed the patient forming a volitional smile (from neutral expression to full smile), which is part of a larger clinical assessment of the patients’ facial function. Patients consisted of 42 females (mean age, 47.74 years; range, 18 to 80) and 19 males (mean age, 50.79 years; range, 24 to 84). The 61 patient videos were selected from 500 patient videos obtained from the Massachusetts Eye and Ear Infirmary. The videos were selected using a multi-step procedure that involved (i) an initial screening to define the patient population, (ii) stratified random sampling to obtain a sample of videos with diverse combinations of facial health, and (iii) quality control checks to remove videos with various issues, as described below.

First, the 500 videos were screened to remove children (<18 years old), post-surgical patients, and patients with severe facial deformities. There were 270 videos (168 female) that met these initial screening criteria. Second, a stratified sampling procedure was used to select 64 videos that spanned a wide variety of facial function. This was done by partitioning the three-dimensional domain of eFACE subscores into 27 mutually exclusive bins (representing all combinations of low, medium, and high on the three eFACE subscores) and then randomly sampling up to three patients from each bin. Third, the videos were subjected to quality control checks, which included an inspection for completeness of recording, image quality, and inclusion of the entire smile function (i.e., all phases of the smile). These quality control checks resulted in the exclusion of three patients (two due to video quality and one due to incomplete smile), which produced our sample of sixty-one patients.

#### 2.2.2. eFACE Scale

Each patient’s degree of facial function was determined by the eFACE scale, which is a clinician-graded scale for assessing facial function in patients with unilateral facial paralysis [32]. The eFACE scale consists of 15 items that combine to form three subscores measuring clinically relevant factors of facial health: Static, Dynamic, and Synkinesis. The Static subscore measures the health of the still (i.e., resting) face, the Dynamic subscore measures the health of the face during voluntary movement tasks (e.g., raise eyebrows, close eyes, etc.), and the Synkinesis subscore measures the health of the face in terms of lack of synkinetic activity (i.e., involuntary movements). The eFACE scale was found to have high inter- and intra-rater reliability [32,35,36], as well as high predictive validity for both expert and layperson perceptions of disfigurement [33,34,37,38].

**Figure 1:**
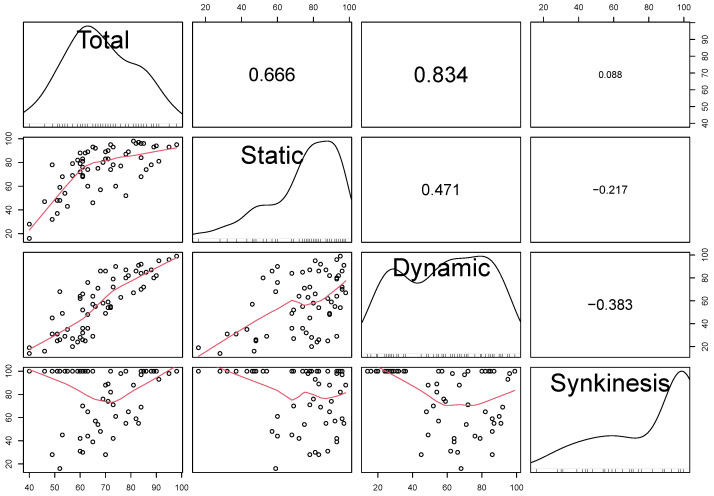
Kernel density estimates showing the sample distribution of the scores (diagonal), scatterplots showing the bivariate associations between the scores (lower triangle), and Pearson correlation between scores (upper triangle).

All of the subscores range from 0 to 100 such that larger values correspond to better facial health. Specifically, “a value of 0 indicated the most extreme malposition (static items), total absence of movement (dynamic items), or the most severe synkinesis (synkinesis items). A value of 100 indicated balanced position (static items), normal movement (dynamic items), or absence of synkinesis (synkinesis items).” (p. 224e of [32]). The eFACE subscores combine to form an eFACE Total score, which ranges from 0 to 100, such that higher values indicate better overall facial function. See Figure 1 for a visualization of the distributions of and associations between the eFACE Total scores.

#### 2.2.3. Interpersonal Adjective Scales-Revised

The Interpersonal Adjective Scales-Revised [39] measure aspects of personality that deal with interacting with others. The IAS-R consists of 64 adjectives that could be used to describe a person, e.g., friendly, cunning, etc. Each item is rated on a Likert-type scale from 1 (extremely inaccurate) to 8 (extremely accurate), indicating how well the given adjective describes a particular individual. The adjectives can be categorized into eight groups (or octants), each consisting of eight adjectives. The octants are theorized to take on the structure of a circumplex, which is embedded within two orthogonal dimensions: Affiliation (i.e., warmth/agreeableness) and Dominance [40,41]. Scores on these two dimensions are obtained by taking a linear combination of the octant scores, which are linear combinations of the item scores [42]. To reduce the burden on study participants, we created a brief version of the scale by selecting two items from each octant, i.e., sixteen items in total, where the two items were selected to have high communalities [41].

### 2.3. Procedure

Raters were provided a tablet (Apple iPad3, 16GB, iOS 8) with a custom-built application to display the patient videos, and a rating sheet to record their responses. The iPad application (i) obtained informed consent from the rater, (ii) provided basic instructions on how to use the app, and then (iii) randomly sampled 1 of the 61 patient videos to display. The rater was asked to both evaluate the patient using 16 adjectives from the IAS-R (see Appendix B) and judge the patient’s Disfigurement, Attractiveness, and Competence (using a 10-point Likert scale). A glossary providing the adjective definitions was available to the raters (see Appendix A and Appendix B). The videos could be replayed as many times as desired while completing the rating sheet, but participants were instructed to rate the videos based on their “gut reaction” to the person in the video. After completing their evaluation of a video, raters could choose to rate more videos (46 participants rated 1 video, 53 participants rated 2 videos, 145 participants rated 3 videos, 294 participants rated 4 videos, and 9 participants rated 5 videos). If the rater chose to rate more than one video, subsequent videos were randomly sampled without replacement such that a rater would not rate the same video more than once.

### 2.4. Analysis

To facilitate comparisons between the results, the Competence rating variable was transformed to the interval [−15,15], which is the approximate range of the Affiliation and Dominance scores. Relating the response variables of interest (i.e., Competence, Affiliation, and Dominance) to the eFACE scores requires a method that can quantify the effects of facial health on the response variables, while accounting for (i) the influence of the covariates, (ii) the correlation between the repeated responses for different raters, and (iii) the correlation between the repeated ratings for different stimuli. We used linear mixed-effects regression (LMER), which allows us to explore the (fixed) effects of facial health on the outcome variables while controlling for the (fixed) effects of the covariates and the (random) effects of the raters and patients. For each response variable, we fit two models: (i) a model including the eFACE total score as the fixed effect predictor and (ii) a model including the additive effects of the three eFACE subscores as fixed effects predictors. For all models, we controlled for the following fixed effects: the rater’s Age and Gender, the patient’s Age and Gender, and the rater’s perceived Attractiveness of the patient. Furthermore, for all models we included random intercepts for both the raters and the patients. The LMER models were fit via the lme4 [43] and lmerTest [44] packages in the R software environment [45]. The R code and data to reproduce our analyses are provided in the Supplementary Materials.

## 3. Results

### 3.1. eFACE Total Score Models

Table 1, Table 2 and Table 3 display the results for the three models that used the eFACE Total score as a predictor. The results in Table 1, Table 2 and Table 3 reveal several interesting effects, and only one effect is consistent across all three response variables: Attractiveness is a significant predictor, such that people who are perceived to be more attractive are also perceived to have more Competence, Affiliation, and Dominance. The other effects differ depending on the response variable. We discuss the results separately for each response variable.

#### 3.1.1. Competence

The results for the Competence ratings are displayed in Table 1. We find that both the patient’s Age and the rater’s Age are significant predictors of the Competence ratings. Older patients are perceived to be more competent, whereas older raters perceive patients to be less competent, which suggests that the relative age of the perceiver to the target may be driving these relationships. The patient’s Gender and the rater’s Gender are not significantly related to the Competence ratings. The rater’s perceived Attractiveness of the patient has the largest effect on the rater’s perceived Competence of the patient, such that patients who are rated to be more attractive are also rated to be more competent. Controlling for the covariates, the patient’s degree of facial health (as measured by eFACE Total) was significantly related to Competence ratings, such that patients with better facial health were rated to be more competent.

**Table 1:** Competence from eFACE Total score: coefficient and variance component information.

Effect	Estimate	SE	95% CI		*p*
			LL	UL	
Fixed effects					
(Intercept)	−7.560	0.792	−9.120	−5.999	0.000
Patient Age	0.051	0.010	0.031	0.070	0.000
Patient Gender ^a^	−0.149	0.338	−0.825	0.527	0.661
Rater Age	−0.045	0.011	−0.066	−0.023	0.000
Rater Gender ^a^	−0.180	0.389	−0.944	0.583	0.643
Attractiveness	1.698	0.070	1.562	1.835	0.000
eFACE Total ^b^	0.032	0.012	0.008	0.057	0.009
Random effects					
Rater	9.259	0.043	7.192	11.392	0.000
Patient	0.495	0.041	0.013	1.026	0.017
Residual	22.333	0.099	20.609	24.273	NA

Note. Number of raters = 538; number of patients = 61; total N=1757; SE = standard error; CI = confidence interval; LL = lower limit; UL = upper limit. R-squared fixed effects only = 0.316; R-squared fixed and random effects = 0.623. ^a^ 0 = Female; 1 = Male. ^b^ Centered across patients.

#### 3.1.2. Affiliation

Table 2 presents the results for the Affiliation rating data. The Affiliation fixed effects results reveal a similar pattern as the Competence results, with one noteworthy exception: we find that male patients are rated to have less Affiliation than female patients. The other fixed effects produce the same interpretation as the Competence results, i.e., (i) older patients are perceived to be more Affiliative, (ii) older raters perceive patients to be less Affiliative, (iii) more Attractive patients are rated to be more Affiliative, and (iv) patients with better facial health were rated to be more Affiliative.

**Table 2:** Affiliation from eFACE Total score: coefficient and variance component information.

Effect	Estimate	SE	95% CI		*p*
			LL	UL	
Fixed effects					
(Intercept)	−4.137	0.813	−5.747	−2.526	0.000
Patient Age	0.056	0.013	0.030	0.083	0.000
Patient Gender ^a^	−1.486	0.450	−2.387	−0.585	0.002
Rater Age	−0.021	0.008	−0.035	−0.006	0.006
Rater Gender ^a^	−0.259	0.271	−0.792	0.274	0.340
Attractiveness	1.060	0.055	0.951	1.169	0.000
eFACE Total ^b^	0.038	0.016	0.006	0.069	0.021
Random effects					
Rater	3.122	0.043	2.141	4.153	0.000
Patient	1.994	0.046	1.135	2.938	0.000
Residual	14.399	0.079	13.279	15.643	NA

Note. Number of raters = 533; number of patients = 61; total N=1718; SE = standard error; CI = confidence interval; LL = lower limit; UL = upper limit. R-squared fixed effects only = 0.253; R-squared fixed and random effects = 0.543. ^a^ 0 = Female; 1 = Male. ^b^ Centered across patients.

#### 3.1.3. Dominance

The results for the Dominance ratings are displayed in Table 3. The patient’s Age and the rater’s Age are not significant predictors. In contrast, the patient’s Gender and the rater’s Gender are significant predictors. Male patients are perceived as more dominant, and male raters perceive patients as more dominant. The rater’s perceived Attractiveness of the patient still has a large effect: more attractive patients are rated to be more dominant. Controlling for the covariates, the patient’s degree of facial health was not significantly related to the perceived Dominance of the patient.

**Table 3:** Dominance from eFACE Total score: coefficient and variance component information.

Effect	Estimate	SE	95% CI		*p*
			LL	UL	
Fixed effects					
(Intercept)	−7.161	0.965	−9.078	−5.244	0.000
Patient Age	0.025	0.017	−0.008	0.059	0.133
Patient Gender ^a^	1.139	0.567	0.003	2.275	0.049
Rater Age	0.004	0.007	−0.010	0.018	0.565
Rater Gender ^a^	0.695	0.263	0.179	1.211	0.008
Attractiveness	0.825	0.056	0.715	0.935	0.000
eFACE Total ^b^	0.000	0.020	−0.040	0.040	0.997
Random effects					
Rater	2.426	0.046	1.464	3.429	0.000
Patient	3.522	0.054	2.155	5.019	0.000
Residual	15.108	0.082	13.925	16.425	NA

Note. Number of raters = 536; number of patients = 61; total N=1725; SE = standard error; CI = confidence interval; LL = lower limit; UL = upper limit. R-squared fixed effects only = 0.126; R-squared fixed and random effects = 0.469. ^a^ 0 = Female; 1 = Male. ^b^ Centered across patients.

### 3.2. eFACE Subscore Models

**Figure 2:**
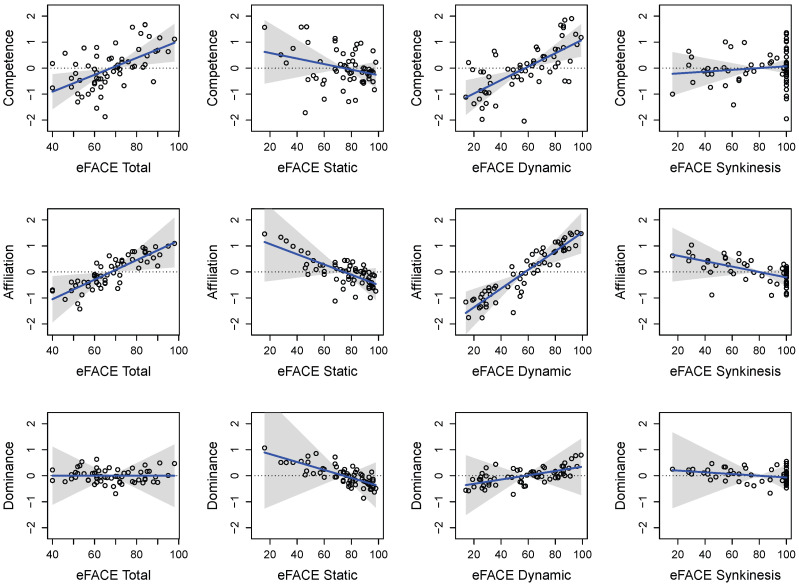
Relationship between first impressions on psychological traits (rows) and overall facial health (**left**), static facial health (**left center**), dynamic facial health (**right center**), and synkinesis facial health (**right**). Points denote the average (across raters) of the model residuals with the plotted effect excluded from the prediction, whereas the blue line denotes the estimated effect. The shadings denote a 95% confidence interval, and the dotted line denotes zero (i.e., no effect).

The results for the eFACE subscores reveal similar patterns as the Total score models with respect to fit information and parameter estimates (see Table A1, Table A2 and Table A3 in Appendix A). As a result, we only comment on aspects of the results which differ. Focusing on the contributions of the eFACE components, the Dynamic subscore is significantly related to Competence and Affiliation ratings, such that patients with better Dynamic facial function are rated to be more competent (see Table A1) and affiliative (see Table A2). In contrast, the patient’s Dynamic subscore is not significantly related to the Dominance ratings (see Table A3). Given the other effects, the eFACE Static and Synkinesis subscores are not significantly related to the patient’s perceived Competence, Affiliation, or Dominance (see Table A1 and Table A2). Thus, the Dynamic subscores seem to be driving the relationships observed in the Total score analysis (see Figure 2).

## 4. Discussion

### 4.1. Summary of Findings

Controlling for well-known effects, our results clearly demonstrate that differences in facial health influence first impressions drawn from faces. First, we established that overall facial health can predict first impressions of Competence and Affiliation, but not Dominance. Then we demonstrated that dynamic facial health is the driving component behind these associations. In other words, dynamic facial health alone predicts both Competence and Affiliation—but not Dominance. Conditioned on dynamic facial health, an individual’s level of static (resting) facial health and synkinesis (involuntary movement) facial health had no significant effects on first impressions of Competence, Affiliation, or Dominance. These findings complement past studies, which have noted that facial dynamics can influence psychological first impressions drawn from healthy faces [46,47,48,49,50].

Consistent with previous work [7,8], we found that patients who were perceived as more attractive were also perceived to have more favorable psychological traits, i.e., more Competence, Affiliation, and Dominance. We also observed that male patients were perceived to be more dominant and less affiliative, which agrees with past findings regarding the effects of facial masculinity on first impressions [20]. We found that older patients were perceived to have higher levels of Competence and Affiliation. These results agree with past findings regarding the effects of facial maturity on Competence first impressions [17] but somewhat disagree with previous results on Affiliation first impressions: Oosterhof and Todorov [20] found that more mature faces were perceived as less trustworthy. However, it should be noted that our result is not directly comparable to the result of Oosterhof and Todorov [20] for two reasons: (a) we used Affiliation as the response, whereas they used Trustworthiness, and (b) we used real videos of patients with different ages, whereas they used computer-generated faces with different (simulated) degrees of facial maturity.

### 4.2. Ecological Interpretation

The idea that facial health can influence first impressions is well-supported by the ecological approach of facial perception. As predicted by ecological theory [1,15], we found evidence that people may overgeneralize deficits in facial health, causing them to perceive negative psychological attributes from unhealthy faces. Note that raters’ perceptions of Disfigurement tend to agree with clinicians’ ratings on the eFACE scale [34], evidencing that raters are, in fact, perceptive of the deficits in patients’ facial health. The importance of dynamic facial health, as compared to static facial health, is also well-supported by the ecological approach, which posits that dynamic functioning provides more information relevant to survival and functioning. This highlights the importance of utilizing dynamic stimuli (e.g., videos or virtual reality) instead of static images when trying to study the affects of facial health on psychological first impressions.

Interestingly, our results suggest that facial health indicators are important for forming judgements about one’s Competence and Affiliation, but not one’s Dominance. Interpreting these findings from the ecological perspective offers some intriguing insights regarding the sorts of first impressions that are obtained from different facial cues. Note that perceptions of Competence and Affiliation are both relevant to choosing reliable leaders and companions [18], making them important for long-term survival and social functioning. In contrast, perceptions of Dominance are relevant for determining whether a person has the potential to be a threat [20], making this trait important for short-term (or immediate) survival and functioning. Consequently, our results suggest that facial health plays a role in the overgeneralization of psychological traits that are relevant to long-term, but not necessarily short-term, survival and functioning.

### 4.3. Limitations and Future Directions

Our research establishes new directions regarding the influence of dynamic facial health on first impressions of psychological traits. The primary limitation of our study is that the fairgoers’ first impressions were based solely on a 3–5 s video of the patient smiling. Future work is needed to determine whether our results generalize to other presentation mediums (e.g., face-to-face interaction) and/or other facial tasks (e.g., having a conversation). Furthermore, although the sample of fairgoers was diverse compared to the WEIRD samples typically used in research [51], future work should validate the results in more diverse samples. Finally, additional research is required to determine whether improving one’s dynamic facial health can improve first impressions, e.g., by obtaining ratings of patients before and after facial surgery. Such extensions could (i) elucidate which aspects of dynamic facial health are most important for forming first impressions, (ii) help guide therapeutic efforts for individuals experiencing issues with facial expression, and (iii) inform both patients and clinicians about the progress of facial rehabilitation efforts.

## 5. Conclusions

This study’s findings, demonstrating that dynamic facial health significantly influences first impressions of competence and affiliation, offer crucial insights for advancing personalized medicine in facial reanimation surgery and rehabilitation. By establishing dynamic facial health as a primary driver of these social perceptions, the research provides a clear target for therapeutic interventions. For instance, understanding that improving dynamic facial health, rather than static features or synkinesis, is key to enhancing positive first impressions can guide surgeons and rehabilitation specialists in prioritizing specific functional outcomes. Future research exploring the impact of facial surgery on first impressions (e.g., pre- and post-operative ratings) can further refine personalized treatment plans, elucidating which aspects of dynamic facial health are most critical for social integration and well-being. This personalized approach, focusing on the dynamic aspects of facial expression, holds the potential to significantly improve the holistic outcomes for individuals undergoing facial reanimation, extending beyond physical restoration to encompass improved social interactions and quality of life.

## Data Availability

The data and R code necessary to reproduce our results are included with the Supplementary Materials that accompany this article.

## Supplementary Materials

The following supporting information can be downloaded at https://www.mdpi.com/article/10.3390/jpm15110530/s1, Dataset: the file “eface-ias-data.csv” is a comma separated values (CSV) file containing the raw data; Report: the file “eface-ias-code.html” is an Rmarkdown generative HTML file that contains all of the R code and output from our analysis.

## Abbreviations

The following abbreviations are used in this manuscript:

IAS	Interpersonal Adjectives Scale
LMER	Linear Mixed-Effects Regression

## Appendix A. Supplementary Tables of Results

**Table A1:** Competence from eFACE subscores: coefficient and variance component information.

Effect	Estimate	SE	95% CI		*p*
			LL	UL	
Fixed effects					
(Intercept)	−7.027	0.826	−8.655	−5.400	0.000
Patient Age	0.040	0.011	0.018	0.062	0.001
Patient Gender ^a^	−0.048	0.339	−0.727	0.631	0.888
Rater Age	−0.044	0.011	−0.065	−0.023	0.000
Rater Gender ^a^	−0.193	0.389	−0.958	0.571	0.620
Attractiveness	1.686	0.070	1.548	1.823	0.000
eFACE Static ^b^	−0.011	0.010	−0.032	0.010	0.309
eFACE Dynamic ^b^	0.026	0.008	0.011	0.041	0.001
eFACE Synkinesis ^b^	0.003	0.007	−0.010	0.017	0.601
Random effects					
Rater	9.299	0.043	7.217	11.427	0.000
Patient	0.428	0.043	0.000	0.870	0.040
Residual	22.316	0.099	20.598	24.261	NA

Note. Number of raters = 538; number of patients = 61; total N=1757; SE = standard error; CI = confidence interval; LL = lower limit; UL = upper limit. R-squared fixed effects only = 0.317; R-squared fixed and random effects = 0.623. ^a^ 0 = Female; 1 = Male. ^b^ Centered across patients.

**Table A2:** Affiliation from eFACE subscores: coefficient and variance component information.

Effect	Estimate	SE	95% CI		*p*
			LL	UL	
Fixed effects					
(Intercept)	−3.397	0.820	−5.023	−1.772	0.000
Patient Age	0.040	0.014	0.013	0.067	0.005
Patient Gender ^a^	−1.133	0.418	−1.971	−0.295	0.009
Rater Age	−0.020	0.008	−0.035	−0.006	0.007
Rater Gender ^a^	−0.273	0.271	−0.805	0.260	0.315
Attractiveness	1.050	0.055	0.941	1.158	0.000
eFACE Static ^b^	−0.020	0.013	−0.046	0.006	0.135
eFACE Dynamic ^b^	0.036	0.009	0.017	0.055	0.000
eFACE Synkinesis ^b^	−0.010	0.008	−0.027	0.006	0.201
Random effects					
Rater	3.116	0.043	2.134	4.143	0.000
Patient	1.512	0.044	0.762	2.181	0.000
Residual	14.404	0.079	13.285	15.650	NA

Note. Number of raters = 533; number of patients = 61; total N=1718; SE = standard error; CI = confidence interval; LL = lower limit; UL = upper limit. R-squared fixed effects only = 0.273; R-squared fixed and random effects = 0.542. ^a^ 0 = Female; 1 = Male. ^b^ Centered across patients.

**Table A3:** Dominance from eFACE subscores: coefficient and variance component information.

Effect	Estimate	SE	95% CI		*p*
			LL	UL	
Fixed effects					
(Intercept)	−6.780	1.069	−8.907	−4.652	0.000
Patient Age	0.017	0.019	−0.021	0.056	0.375
Patient Gender ^a^	1.220	0.591	0.037	2.404	0.043
Rater Age	0.004	0.007	−0.010	0.019	0.560
Rater Gender ^a^	0.693	0.263	0.177	1.210	0.009
Attractiveness	0.824	0.056	0.713	0.934	0.000
eFACE Static ^b^	−0.015	0.018	−0.052	0.021	0.409
eFACE Dynamic ^b^	0.008	0.013	−0.018	0.034	0.533
eFACE Synkinesis ^b^	−0.003	0.011	−0.026	0.020	0.772
Random effects					
Rater	2.434	0.046	1.469	3.436	0.000
Patient	3.605	0.056	2.113	4.931	0.000
Residual	15.102	0.082	13.921	16.420	NA

Note. Number of raters = 536; number of patients = 61; total N=1725; SE = standard error; CI = confidence interval; LL = lower limit; UL = upper limit. R-squared fixed effects only = 0.128; R-squared fixed and random effects = 0.469. ^a^ 0 = Female; 1 = Male. ^b^ Centered across patients.

## Appendix B. Interpersonal Adjectives Glossary

1. Assertive: tends to be aggressive and outspoken with others.
2. Coldhearted: have little warmth or feelings for others; unfeeling; harsh.
3. Cunning: crafty, skillful at manipulating others, devious.
4. Friendly: open, accepting, warm around others.
5. Gentle-hearted: warm or kind to others.
6. Introverted: enjoys being alone, reserved around others.
7. Outgoing: enjoy meeting other people.
8. Self-confident: sure of oneself around others, devious.
9. Shy: lacking in self-confidence; tends to be uncomfortable around others.
10. Sly: crafty, secretive, or cunning in dealing with others.
11. Tender-hearted: having a kind, gentle or sentimental nature.
12. Timid: tends to be fearful or uncomfortable around others.
13. Uncrafty: not tricky or sly when dealing with others.
14. Uncunning: not crafty or sly, tends to be straightforward with others.
15. Unsociable: doesn’t enjoy meeting people or being in the company of others.
16. Unsympathetic: not interested or concerned about others’ feelings or problems.
