# Supplementary material for: Dynamic Facial Health Predicts Psychological First Impressions with Applications to Tailored Treatments for Facial Paralysis
Source: J Pers Med. 2025 Nov 2;15(11):530. doi: 10.3390/jpm15110530 (PMC12653328; doi:10.3390/jpm15110530)
Supplement: Supplementary file 1 [file jpm-15-00530-s001.zip › eface-ias-code.html]

eface-ias-code.knit


# Supplementary Materials (R Code) for: Dynamic Facial Health Predicts First Impressions

#### Nathaniel E. Helwig, Lauren N. Berry, Tessa A. Hadlock, Stephen J. Guy, and Sofía Lyford-Pike

#### July 21, 2025

- 1 Overview
- 2 Load and Preprocess Data
  - 2.1 Load the Data
  - 2.2 Remove Some Raters
  - 2.3 Rescale Competence
    Ratings
  - 2.4 Center eFACE Scores
- 3 Demographic Information
  - 3.1 Rater Demographics
  - 3.2 Patient Demographics
- 4 Figure 1: eFACE Scatterplot
  Matrix
- 5 eFACE Score Distributions
  - 5.1 eFACE Total Score
  - 5.2 eFACE Static Score
  - 5.3 eFACE Dynamic Score
  - 5.4 eFACE Synkinesis Score
- 6 eFACE Total Score Correlations
  - 6.1 Total and Static
  - 6.2 Total and Dynamic
  - 6.3 Total and Synkinesis
- 7 eFACE Subscore Correlations
  - 7.1 Static and Dynamic
  - 7.2 Static and Synkinesis
  - 7.3 Dynamic and Synkinesis
- 8 IAS-R Dominance and
  Affiliation
- 9 eFACE Total Score Analyses
  - 9.1 Overview
  - 9.2 Competence
  - 9.3 Affiliation
  - 9.4 Dominance
- 10 eFACE Combined Subscore
  Analyses
  - 10.1 Competence
  - 10.2 Affiliation
  - 10.3 Dominance
- 11 Table Results
  - 11.1 Function to Table
    Results
  - 11.2 Table 1: Competence ~ eFACE
    Total
  - 11.3 Table 2: Affiliation ~ eFACE
    Total
  - 11.4 Table 3: Dominance ~ eFACE
    Total
  - 11.5 Table 4: Competence ~ eFACE
    Subscores
  - 11.6 Table 5: Affiliation ~ eFACE
    Subscores
  - 11.7 Table 6: Dominance ~ eFACE
    Subscores
- 12 Figure 2: eFACE Conditional
  Effects
- 13 Power Analyses
  - 13.1 lmer.power Function
  - 13.2 eFACE Total Score Power
    - 13.2.1 Competence
    - 13.2.2 Affiliation
    - 13.2.3 Dominance
    - 13.2.4 Table Results
  - 13.3 eFACE Subscore Power
    - 13.3.1 Competence
    - 13.3.2 Affiliation
    - 13.3.3 Dominance
    - 13.3.4 Table Results

# 1 Overview

This (R Markdown generated) document contains the R code to replicate
the results in

Dynamic facial health predicts first impressions.

The dataset “eface-ias-data.csv” is obtainable from the Supplementary
Materials.

# 2 Load and Preprocess Data

## 2.1 Load the Data

```
sfdata <- read.csv("eface-ias-data.csv")
dim(sfdata)
```

```
## [1] 1839   32
```

Note that the dataset has \(n =
1839\) observations and \(p =
32\) variables:

```
names(sfdata)
```

```
##  [1] "videoID"        "videoAge"       "videoGender"    "Total"         
##  [5] "Static"         "Dynamic"        "Synkinesis"     "raterID"       
##  [9] "raterAge"       "raterGender"    "raterDrinks"    "Attractiveness"
## [13] "Competence"     "Disfigurement"  "Assertive"      "SelfConfident" 
## [17] "Shy"            "Timid"          "Cunning"        "Sly"           
## [21] "Uncrafty"       "Uncunning"      "Coldhearted"    "Unsympathetic" 
## [25] "Gentlehearted"  "Tenderhearted"  "Introverted"    "Unsociable"    
## [29] "Friendly"       "Outgoing"       "Affiliation"    "Dominance"
```

See the data documentation (i.e., README file) for details on the
variables.

## 2.2 Remove Some Raters

Before analysis, we will remove any raters that reported consuming
three or more alcoholic drinks:

```
sfdata <- subset(sfdata, raterDrinks < 3L)
dim(sfdata)
```

```
## [1] 1813   32
```

Note that we now have \(n = 1813\)
observations left, i.e., we removed 26 observations.

We will also remove the three raters who identified with a gender of
“Other”, given that three observations is not enough to provide
realiable effect estimates:

```
sfdata <- subset(sfdata, raterGender %in% c("Female", "Male"))
dim(sfdata)
```

```
## [1] 1808   32
```

Finally, we will use the function to drop the unused factor levels
for the rater IDs and Gender:

```
sfdata$raterID <- droplevels(as.factor(sfdata$raterID))
sfdata$raterGender <- droplevels(as.factor(sfdata$raterGender))
```

## 2.3 Rescale Competence Ratings

The range of the Competence ratings (i.e., 1 to 10) is rather
different than the range of the Affiliation and Dominance scores (\(\approx\) -15 to 15).

```
range(sfdata$Competence, na.rm = TRUE)
```

```
## [1]  1 10
```

```
range(sfdata$Affiliation, na.rm = TRUE)
```

```
## [1] -13.01041  15.48528
```

```
range(sfdata$Dominance, na.rm = TRUE)
```

```
## [1] -15.39949  14.42462
```

As a result, we rescaled the Competence ratings so the three
variables were on a similar scale.

```
sfdata$Competence <- (sfdata$Competence - 5.5) * (10/3)
range(sfdata$Competence, na.rm = TRUE)
```

```
## [1] -15  15
```

## 2.4 Center eFACE Scores

The eFACE scores were mean-centered prior to fitting the regression
models.

First, we will subset the dataset to obtain the unique patients’ data
(without the raters’ data).

```
patient.vars <- c("videoID", "videoAge", "videoGender", 
                  "Total", "Static", "Dynamic", "Synkinesis")
uniPatients <- unique(sfdata[,patient.vars])
rownames(uniPatients) <- 1:nrow(uniPatients)
dim(uniPatients)
```

```
## [1] 61  7
```

Note that there are 61 unique patients.

Next, we will get the mean of each eFACE score (total and subscores)
across the 61 patients.

```
TotalMean <- mean(uniPatients$Total)
StaticMean <- mean(uniPatients$Static)
DynamicMean <- mean(uniPatients$Dynamic)
SynkinesisMean <- mean(uniPatients$Synkinesis)
```

Finally, we will use the variable means to center the eFACE
scores:

```
sfdata$Total <- sfdata$Total - TotalMean
sfdata$Static <- sfdata$Static - StaticMean
sfdata$Dynamic <- sfdata$Dynamic - DynamicMean
sfdata$Synkinesis <- sfdata$Synkinesis - SynkinesisMean
```

The resulting (centered) scores will be equal to zero if the patient
has “average” facial health, positive if the patient has above-average
facial health, and negative if the patient has below-average facial
health.

# 3 Demographic Information

## 3.1 Rater Demographics

First, we will subset the dataset to obtain the unique raters’ data
(without the patients’ data).

```
uniRaters <- unique(sfdata[, c("raterID", "raterAge", "raterGender")])
dim(uniRaters)
```

```
## [1] 547   3
```

Note that there are 547 unique raters (after the subsetting).

Next, we will tabulate the Gender of the raters:

```
with(uniRaters, table(raterGender))
```

```
## raterGender
## Female   Male 
##    383    164
```

Note that there are 383 Female raters and 164 Male raters.

Lastly, we will get the average, standard deviation (SD), and range
of the rater Ages separately by Gender.

```
with(uniRaters, tapply(raterAge, raterGender, mean))
```

```
##   Female     Male 
## 40.01044 39.25000
```

```
with(uniRaters, tapply(raterAge, raterGender, sd))
```

```
##   Female     Male 
## 16.36382 16.33783
```

```
with(uniRaters, tapply(raterAge, raterGender, range))
```

```
## $Female
## [1] 18 80
## 
## $Male
## [1] 18 86
```

## 3.2 Patient Demographics

First, we will tabulate the Gender of the patients:

```
with(uniPatients, table(videoGender))
```

```
## videoGender
## Female   Male 
##     42     19
```

Note that there are 42 Female patients and 19 Male patients.

Next, we will get the average, standard deviation (SD), and range of
the patient Ages separately by Gender.

```
with(uniPatients, tapply(videoAge, videoGender, mean))
```

```
##   Female     Male 
## 47.73810 50.78947
```

```
with(uniPatients, tapply(videoAge, videoGender, sd))
```

```
##   Female     Male 
## 15.06904 19.36658
```

```
with(uniPatients, tapply(videoAge, videoGender, range))
```

```
## $Female
## [1] 18 80
## 
## $Male
## [1] 24 84
```

# 4 Figure 1: eFACE Scatterplot Matrix

Syntax for Figure 1:

```
# panel function for kernel density estimate
panel.den <- function(x, col = "black", rug = TRUE, 
                      xlim = NULL, ...){
  kde <- density(x)
  usr <- par("usr")
  on.exit(par(usr))
  par(usr = c(usr[1:2], 0, 1.5))
  par(new = TRUE)
  if(is.null(xlim)) xlim <- range(x)
  plot(kde, col = col, main = "", xlim = xlim, axes = FALSE)
  box()
  if(rug) rug(x)
}

# panel function for correlation calculation
panel.cor <- function(x, y, digits = 3, ...){
  rval <- round(cor(x, y), digits)
  usr <- par("usr")
  x <- mean(usr[1:2])
  y <- mean(usr[3:4])
  size <- 2 * abs(rval) + 1
  text(x, y, labels = rval, cex = size)
}

# scatterplot matrix using pairs function
par(mar = c(5, 5, 5, 2))
pairs(uniPatients[,4:7], lower.panel = panel.smooth,
      upper.panel = panel.cor, diag.panel = panel.den,
      cex.labels = 3)
```

```
## Warning in par(usr): argument 1 does not name a graphical parameter
## Warning in par(usr): argument 1 does not name a graphical parameter
## Warning in par(usr): argument 1 does not name a graphical parameter
## Warning in par(usr): argument 1 does not name a graphical parameter
```

```
dev.copy2pdf(file = "fig1.pdf")
```

```
## quartz_off_screen 
##                 2
```

# 5 eFACE Score Distributions

## 5.1 eFACE Total Score

Range, mean, and SD of eFACE Total scores:

```
range(uniPatients$Total)
```

```
## [1] 40 98
```

```
mean(uniPatients$Total)
```

```
## [1] 67.91803
```

```
sd(uniPatients$Total)
```

```
## [1] 13.74808
```

## 5.2 eFACE Static Score

Range, mean, and SD of eFACE Static scores:

```
range(uniPatients$Static)
```

```
## [1] 16 98
```

```
mean(uniPatients$Static)
```

```
## [1] 74.4918
```

```
sd(uniPatients$Static)
```

```
## [1] 19.36029
```

## 5.3 eFACE Dynamic Score

Range, mean, and SD of eFACE Dynamic scores:

```
range(uniPatients$Dynamic)
```

```
## [1] 14 99
```

```
mean(uniPatients$Dynamic)
```

```
## [1] 57.80328
```

```
sd(uniPatients$Dynamic)
```

```
## [1] 24.79504
```

## 5.4 eFACE Synkinesis Score

Range, mean, and SD of eFACE Synkinsesis scores:

```
range(uniPatients$Synkinesis)
```

```
## [1]  16 100
```

```
mean(uniPatients$Synkinesis)
```

```
## [1] 79.62295
```

```
sd(uniPatients$Synkinesis)
```

```
## [1] 25.65487
```

# 6 eFACE Total Score Correlations

## 6.1 Total and Static

```
with(uniPatients, cor.test(Total, Static))
```

```
## 
##  Pearson's product-moment correlation
## 
## data:  Total and Static
## t = 6.8607, df = 59, p-value = 4.654e-09
## alternative hypothesis: true correlation is not equal to 0
## 95 percent confidence interval:
##  0.4978455 0.7861033
## sample estimates:
##       cor 
## 0.6661522
```

## 6.2 Total and Dynamic

```
with(uniPatients, cor.test(Total, Dynamic))
```

```
## 
##  Pearson's product-moment correlation
## 
## data:  Total and Dynamic
## t = 11.619, df = 59, p-value < 2.2e-16
## alternative hypothesis: true correlation is not equal to 0
## 95 percent confidence interval:
##  0.7372587 0.8974891
## sample estimates:
##       cor 
## 0.8342037
```

## 6.3 Total and Synkinesis

Using all 61 patients:

```
with(uniPatients, cor.test(Total, Synkinesis))
```

```
## 
##  Pearson's product-moment correlation
## 
## data:  Total and Synkinesis
## t = 0.67851, df = 59, p-value = 0.5001
## alternative hypothesis: true correlation is not equal to 0
## 95 percent confidence interval:
##  -0.1675411  0.3324463
## sample estimates:
##        cor 
## 0.08799201
```

Using the 32 patients with some form of synkinesis:

```
with(subset(uniPatients, Synkinesis < 100), cor.test(Total, Synkinesis))
```

```
## 
##  Pearson's product-moment correlation
## 
## data:  Total and Synkinesis
## t = 5.2426, df = 30, p-value = 1.174e-05
## alternative hypothesis: true correlation is not equal to 0
## 95 percent confidence interval:
##  0.4516687 0.8380859
## sample estimates:
##       cor 
## 0.6914615
```

# 7 eFACE Subscore Correlations

## 7.1 Static and Dynamic

Using all 61 patients:

```
with(uniPatients, cor.test(Static, Dynamic))
```

```
## 
##  Pearson's product-moment correlation
## 
## data:  Static and Dynamic
## t = 4.0989, df = 59, p-value = 0.0001286
## alternative hypothesis: true correlation is not equal to 0
## 95 percent confidence interval:
##  0.2484226 0.6460230
## sample estimates:
##       cor 
## 0.4707916
```

Using the 42 patients with moderate-to-high dynamic function:

```
with(subset(uniPatients, Dynamic > 40), cor.test(Static, Dynamic))
```

```
## 
##  Pearson's product-moment correlation
## 
## data:  Static and Dynamic
## t = 0.48679, df = 40, p-value = 0.6291
## alternative hypothesis: true correlation is not equal to 0
## 95 percent confidence interval:
##  -0.2326156  0.3719962
## sample estimates:
##        cor 
## 0.07674132
```

Using the 19 patients with low dynamic function:

```
with(subset(uniPatients, Dynamic < 40), cor.test(Static, Dynamic))
```

```
## 
##  Pearson's product-moment correlation
## 
## data:  Static and Dynamic
## t = 1.1329, df = 17, p-value = 0.273
## alternative hypothesis: true correlation is not equal to 0
## 95 percent confidence interval:
##  -0.2151487  0.6419116
## sample estimates:
##       cor 
## 0.2649524
```

## 7.2 Static and Synkinesis

Using the 32 patients with some form of synkinesis:

```
with(subset(uniPatients, Synkinesis < 100), cor.test(Static, Synkinesis))
```

```
## 
##  Pearson's product-moment correlation
## 
## data:  Static and Synkinesis
## t = 2.1239, df = 30, p-value = 0.04204
## alternative hypothesis: true correlation is not equal to 0
## 95 percent confidence interval:
##  0.01469186 0.63071662
## sample estimates:
##       cor 
## 0.3615337
```

## 7.3 Dynamic and Synkinesis

Using the 32 patients with some form of synkinesis:

```
with(subset(uniPatients, Synkinesis < 100), cor.test(Dynamic, Synkinesis))
```

```
## 
##  Pearson's product-moment correlation
## 
## data:  Dynamic and Synkinesis
## t = 0.19102, df = 30, p-value = 0.8498
## alternative hypothesis: true correlation is not equal to 0
## 95 percent confidence interval:
##  -0.3177008  0.3789429
## sample estimates:
##        cor 
## 0.03485438
```

# 8 IAS-R Dominance and Affiliation

The Dominance and Affiliation scores are linear combinations of the
16 item adjective ratings, which can be verified using the following R
code:

```
# octant scores
PA <- with(sfdata, SelfConfident + Assertive) / 2
BC <- with(sfdata, Sly + Cunning) / 2
DE <- with(sfdata, Coldhearted + Unsympathetic) / 2
FG <- with(sfdata, Introverted + Unsociable) / 2
HI <- with(sfdata, Timid + Shy) / 2
JK <- with(sfdata, Uncunning + Uncrafty) / 2
LM <- with(sfdata, Gentlehearted + Tenderhearted) / 2
NO <- with(sfdata, Outgoing + Friendly) / 2

# make dominance and affiliation
DOM <- PA - HI + (sqrt(2)/2) * (NO + BC - FG - JK)
AFF <- LM - DE + (sqrt(2)/2) * (NO - BC - FG + JK)

# confirm these are identical to those in 'sfdata'
all.equal(DOM, sfdata$Dominance)
```

```
## [1] TRUE
```

```
all.equal(AFF, sfdata$Affiliation)
```

```
## [1] TRUE
```

# 9 eFACE Total Score Analyses

## 9.1 Overview

Load the **lme4** and **lmerTest** packages
in R.

```
library(lme4)        # version:  1.1-37
library(lmerTest)    # version:  3.1-3
```

## 9.2 Competence

Predict Competence from eFACE Total score (and covariates):

```
form <- formula(Competence ~ videoAge + videoGender + raterAge + 
                  raterGender + Attractiveness + Total + 
                  (1 | raterID) + (1 | videoID))
total.Com <- lmer(formula = form, data = sfdata)
total.Com.sum <- summary(total.Com)
total.Com.sum
```

```
## Linear mixed model fit by REML. t-tests use Satterthwaite's method [
## lmerModLmerTest]
## Formula: form
##    Data: sfdata
## 
## REML criterion at convergence: 10940.1
## 
## Scaled residuals: 
##     Min      1Q  Median      3Q     Max 
## -3.5268 -0.5588  0.0369  0.5570  3.5799 
## 
## Random effects:
##  Groups   Name        Variance Std.Dev.
##  raterID  (Intercept)  9.2592  3.0429  
##  videoID  (Intercept)  0.4945  0.7032  
##  Residual             22.3328  4.7258  
## Number of obs: 1757, groups:  raterID, 538; videoID, 61
## 
## Fixed effects:
##                   Estimate Std. Error         df t value Pr(>|t|)    
## (Intercept)     -7.560e+00  7.923e-01  2.561e+02  -9.542  < 2e-16 ***
## videoAge         5.064e-02  9.918e-03  5.844e+01   5.106 3.77e-06 ***
## videoGenderMale -1.490e-01  3.380e-01  5.873e+01  -0.441  0.66097    
## raterAge        -4.464e-02  1.082e-02  5.002e+02  -4.128 4.30e-05 ***
## raterGenderMale -1.804e-01  3.887e-01  5.064e+02  -0.464  0.64277    
## Attractiveness   1.698e+00  6.973e-02  1.437e+03  24.358  < 2e-16 ***
## Total            3.248e-02  1.202e-02  6.129e+01   2.702  0.00891 ** 
## ---
## Signif. codes:  0 '***' 0.001 '**' 0.01 '*' 0.05 '.' 0.1 ' ' 1
## 
## Correlation of Fixed Effects:
##             (Intr) videAg vdGndM ratrAg rtrGnM Attrct
## videoAge    -0.666                                   
## videoGndrMl -0.137 -0.051                            
## raterAge    -0.495 -0.005 -0.007                     
## raterGndrMl -0.212  0.005  0.032  0.015              
## Attractvnss -0.496  0.148  0.085 -0.103  0.119       
## Total       -0.066  0.233  0.057  0.019 -0.032 -0.200
```

## 9.3 Affiliation

Predict Affiliation from eFACE Total score (and covariates):

```
form <- formula(Affiliation ~ videoAge + videoGender + raterAge + 
                  raterGender + Attractiveness + Total + 
                  (1 | raterID) + (1 | videoID))
total.Aff <- lmer(formula = form, data = sfdata)
total.Aff.sum <- summary(total.Aff)
total.Aff.sum
```

```
## Linear mixed model fit by REML. t-tests use Satterthwaite's method [
## lmerModLmerTest]
## Formula: form
##    Data: sfdata
## 
## REML criterion at convergence: 9845.4
## 
## Scaled residuals: 
##     Min      1Q  Median      3Q     Max 
## -3.5730 -0.5632  0.0383  0.5936  3.3888 
## 
## Random effects:
##  Groups   Name        Variance Std.Dev.
##  raterID  (Intercept)  3.122   1.767   
##  videoID  (Intercept)  1.994   1.412   
##  Residual             14.399   3.795   
## Number of obs: 1718, groups:  raterID, 533; videoID, 61
## 
## Fixed effects:
##                   Estimate Std. Error         df t value Pr(>|t|)    
## (Intercept)       -4.13684    0.81283  112.29180  -5.089 1.46e-06 ***
## videoAge           0.05641    0.01323   57.68314   4.264 7.54e-05 ***
## videoGenderMale   -1.48608    0.45005   57.77486  -3.302  0.00165 ** 
## raterAge          -0.02071    0.00752  508.08202  -2.753  0.00611 ** 
## raterGenderMale   -0.25888    0.27126  517.05097  -0.954  0.34034    
## Attractiveness     1.05995    0.05537 1616.52118  19.142  < 2e-16 ***
## Total              0.03763    0.01588   58.68287   2.369  0.02115 *  
## ---
## Signif. codes:  0 '***' 0.001 '**' 0.01 '*' 0.05 '.' 0.1 ' ' 1
## 
## Correlation of Fixed Effects:
##             (Intr) videAg vdGndM ratrAg rtrGnM Attrct
## videoAge    -0.811                                   
## videoGndrMl -0.139 -0.060                            
## raterAge    -0.324 -0.002 -0.007                     
## raterGndrMl -0.155  0.003  0.020  0.014              
## Attractvnss -0.380  0.087  0.051 -0.124  0.138       
## Total       -0.178  0.256  0.066  0.010 -0.023 -0.110
```

## 9.4 Dominance

Predict Dominance from eFACE Total score (and covariates):

```
form <- formula(Dominance ~ videoAge + videoGender + raterAge + 
                  raterGender + Attractiveness + Total + 
                  (1 | raterID) + (1 | videoID))
total.Dom <- lmer(formula = form, data = sfdata)
total.Dom.sum <- summary(total.Dom)
total.Dom.sum
```

```
## Linear mixed model fit by REML. t-tests use Satterthwaite's method [
## lmerModLmerTest]
## Formula: form
##    Data: sfdata
## 
## REML criterion at convergence: 9934.7
## 
## Scaled residuals: 
##     Min      1Q  Median      3Q     Max 
## -2.8537 -0.6208  0.0000  0.6411  3.2601 
## 
## Random effects:
##  Groups   Name        Variance Std.Dev.
##  raterID  (Intercept)  2.426   1.558   
##  videoID  (Intercept)  3.522   1.877   
##  Residual             15.108   3.887   
## Number of obs: 1725, groups:  raterID, 536; videoID, 61
## 
## Fixed effects:
##                   Estimate Std. Error         df t value Pr(>|t|)    
## (Intercept)     -7.161e+00  9.648e-01  8.991e+01  -7.422 6.24e-11 ***
## videoAge         2.542e-02  1.668e-02  5.772e+01   1.525  0.13282    
## videoGenderMale  1.139e+00  5.674e-01  5.790e+01   2.008  0.04935 *  
## raterAge         4.180e-03  7.262e-03  4.836e+02   0.576  0.56517    
## raterGenderMale  6.950e-01  2.627e-01  4.958e+02   2.645  0.00842 ** 
## Attractiveness   8.251e-01  5.605e-02  1.567e+03  14.722  < 2e-16 ***
## Total           -6.512e-05  1.998e-02  5.828e+01  -0.003  0.99741    
## ---
## Signif. codes:  0 '***' 0.001 '**' 0.01 '*' 0.05 '.' 0.1 ' ' 1
## 
## Correlation of Fixed Effects:
##             (Intr) videAg vdGndM ratrAg rtrGnM Attrct
## videoAge    -0.850                                   
## videoGndrMl -0.142 -0.062                            
## raterAge    -0.261 -0.001 -0.005                     
## raterGndrMl -0.128  0.001  0.018  0.011              
## Attractvnss -0.326  0.070  0.040 -0.128  0.146       
## Total       -0.207  0.259  0.069  0.010 -0.019 -0.087
```

# 10 eFACE Combined Subscore Analyses

## 10.1 Competence

Predict Competence from eFACE Subscores (and covariates):

```
form <- formula(Competence ~ videoAge + videoGender + raterAge + 
                  raterGender + Attractiveness + Static + Dynamic + 
                  Synkinesis + (1 | raterID) + (1 | videoID))
sub.Com <- lmer(formula = form, data = sfdata)
sub.Com.sum <- summary(sub.Com)
sub.Com.sum
```

```
## Linear mixed model fit by REML. t-tests use Satterthwaite's method [
## lmerModLmerTest]
## Formula: form
##    Data: sfdata
## 
## REML criterion at convergence: 10952.3
## 
## Scaled residuals: 
##     Min      1Q  Median      3Q     Max 
## -3.5021 -0.5580  0.0403  0.5492  3.6275 
## 
## Random effects:
##  Groups   Name        Variance Std.Dev.
##  raterID  (Intercept)  9.2989  3.0494  
##  videoID  (Intercept)  0.4282  0.6544  
##  Residual             22.3155  4.7239  
## Number of obs: 1757, groups:  raterID, 538; videoID, 61
## 
## Fixed effects:
##                   Estimate Std. Error         df t value Pr(>|t|)    
## (Intercept)     -7.027e+00  8.256e-01  2.135e+02  -8.512 3.04e-15 ***
## videoAge         4.017e-02  1.099e-02  5.408e+01   3.654 0.000584 ***
## videoGenderMale -4.788e-02  3.388e-01  5.541e+01  -0.141 0.888151    
## raterAge        -4.420e-02  1.083e-02  4.992e+02  -4.082 5.20e-05 ***
## raterGenderMale -1.932e-01  3.891e-01  5.056e+02  -0.497 0.619701    
## Attractiveness   1.686e+00  7.024e-02  1.502e+03  23.998  < 2e-16 ***
## Static          -1.068e-02  1.039e-02  5.203e+01  -1.028 0.308803    
## Dynamic          2.587e-02  7.545e-03  5.700e+01   3.428 0.001135 ** 
## Synkinesis       3.436e-03  6.533e-03  5.447e+01   0.526 0.601048    
## ---
## Signif. codes:  0 '***' 0.001 '**' 0.01 '*' 0.05 '.' 0.1 ' ' 1
## 
## Correlation of Fixed Effects:
##             (Intr) videAg vdGndM ratrAg rtrGnM Attrct Static Dynamc
## videoAge    -0.699                                                 
## videoGndrMl -0.102 -0.073                                          
## raterAge    -0.471 -0.011 -0.001                                   
## raterGndrMl -0.205  0.005  0.025  0.015                            
## Attractvnss -0.486  0.152  0.052 -0.104  0.121                     
## Static      -0.311  0.510 -0.026 -0.008 -0.011 -0.031              
## Dynamic      0.170 -0.170  0.095  0.025 -0.027 -0.184 -0.421       
## Synkinesis  -0.059  0.113 -0.167 -0.009  0.013  0.025  0.100  0.276
```

## 10.2 Affiliation

Predict Affiliation from eFACE Subscores (and covariates):

```
form <- formula(Affiliation ~ videoAge + videoGender + raterAge + 
                  raterGender + Attractiveness + Static + Dynamic + 
                  Synkinesis + (1 | raterID) + (1 | videoID))
sub.Aff <- lmer(formula = form, data = sfdata)
sub.Aff.sum <- summary(sub.Aff)
sub.Aff.sum
```

```
## Linear mixed model fit by REML. t-tests use Satterthwaite's method [
## lmerModLmerTest]
## Formula: form
##    Data: sfdata
## 
## REML criterion at convergence: 9847.6
## 
## Scaled residuals: 
##     Min      1Q  Median      3Q     Max 
## -3.5427 -0.5689  0.0388  0.5973  3.3645 
## 
## Random effects:
##  Groups   Name        Variance Std.Dev.
##  raterID  (Intercept)  3.116   1.765   
##  videoID  (Intercept)  1.512   1.230   
##  Residual             14.404   3.795   
## Number of obs: 1718, groups:  raterID, 533; videoID, 61
## 
## Fixed effects:
##                   Estimate Std. Error         df t value Pr(>|t|)    
## (Intercept)     -3.397e+00  8.197e-01  1.053e+02  -4.145 6.89e-05 ***
## videoAge         4.004e-02  1.362e-02  5.466e+01   2.940  0.00480 ** 
## videoGenderMale -1.133e+00  4.182e-01  5.517e+01  -2.710  0.00896 ** 
## raterAge        -2.048e-02  7.513e-03  5.075e+02  -2.725  0.00664 ** 
## raterGenderMale -2.728e-01  2.711e-01  5.165e+02  -1.006  0.31466    
## Attractiveness   1.050e+00  5.532e-02  1.610e+03  18.972  < 2e-16 ***
## Static          -1.968e-02  1.296e-02  5.409e+01  -1.519  0.13466    
## Dynamic          3.600e-02  9.283e-03  5.566e+01   3.878  0.00028 ***
## Synkinesis      -1.045e-02  8.081e-03  5.471e+01  -1.294  0.20127    
## ---
## Signif. codes:  0 '***' 0.001 '**' 0.01 '*' 0.05 '.' 0.1 ' ' 1
## 
## Correlation of Fixed Effects:
##             (Intr) videAg vdGndM ratrAg rtrGnM Attrct Static Dynamc
## videoAge    -0.826                                                 
## videoGndrMl -0.101 -0.082                                          
## raterAge    -0.319 -0.006 -0.004                                   
## raterGndrMl -0.152  0.002  0.016  0.014                            
## Attractvnss -0.379  0.091  0.034 -0.124  0.138                     
## Static      -0.410  0.524 -0.033 -0.003 -0.010 -0.024              
## Dynamic      0.149 -0.164  0.106  0.014 -0.019 -0.106 -0.434       
## Synkinesis  -0.061  0.104 -0.171 -0.008  0.008  0.018  0.093  0.279
```

## 10.3 Dominance

Predict Dominance from eFACE Subscores (and covariates):

```
form <- formula(Dominance ~ videoAge + videoGender + raterAge + 
                  raterGender + Attractiveness + Static + Dynamic + 
                  Synkinesis + (1 | raterID) + (1 | videoID))
sub.Dom <- lmer(formula = form, data = sfdata)
sub.Dom.sum <- summary(sub.Dom)
sub.Dom.sum
```

```
## Linear mixed model fit by REML. t-tests use Satterthwaite's method [
## lmerModLmerTest]
## Formula: form
##    Data: sfdata
## 
## REML criterion at convergence: 9948.3
## 
## Scaled residuals: 
##     Min      1Q  Median      3Q     Max 
## -2.8454 -0.6206 -0.0008  0.6403  3.2646 
## 
## Random effects:
##  Groups   Name        Variance Std.Dev.
##  raterID  (Intercept)  2.434   1.560   
##  videoID  (Intercept)  3.605   1.899   
##  Residual             15.102   3.886   
## Number of obs: 1725, groups:  raterID, 536; videoID, 61
## 
## Fixed effects:
##                   Estimate Std. Error         df t value Pr(>|t|)    
## (Intercept)     -6.780e+00  1.069e+00  7.889e+01  -6.344 1.31e-08 ***
## videoAge         1.720e-02  1.924e-02  5.521e+01   0.894  0.37516    
## videoGenderMale  1.220e+00  5.906e-01  5.560e+01   2.067  0.04344 *  
## raterAge         4.241e-03  7.266e-03  4.832e+02   0.584  0.55966    
## raterGenderMale  6.934e-01  2.629e-01  4.955e+02   2.637  0.00861 ** 
## Attractiveness   8.236e-01  5.617e-02  1.562e+03  14.662  < 2e-16 ***
## Static          -1.527e-02  1.835e-02  5.506e+01  -0.832  0.40874    
## Dynamic          8.220e-03  1.309e-02  5.575e+01   0.628  0.53269    
## Synkinesis      -3.318e-03  1.142e-02  5.523e+01  -0.291  0.77247    
## ---
## Signif. codes:  0 '***' 0.001 '**' 0.01 '*' 0.05 '.' 0.1 ' ' 1
## 
## Correlation of Fixed Effects:
##             (Intr) videAg vdGndM ratrAg rtrGnM Attrct Static Dynamc
## videoAge    -0.879                                                 
## videoGndrMl -0.104 -0.084                                          
## raterAge    -0.234 -0.004 -0.002                                   
## raterGndrMl -0.115  0.000  0.013  0.010                            
## Attractvnss -0.295  0.065  0.024 -0.129  0.147                     
## Static      -0.452  0.528 -0.034 -0.003 -0.006 -0.019              
## Dynamic      0.143 -0.165  0.110  0.012 -0.015 -0.073 -0.440       
## Synkinesis  -0.061  0.101 -0.175 -0.005  0.008  0.013  0.090  0.279
```

# 11 Table Results

## 11.1 Function to Table Results

Define R function to make APA style table for mixed effects
models

```
apatab <- function(object, level = 0.95, digits = 3){
  # object must be of class "lmerModLmerTest"
  
  # significance level and summary
  alpha <- 1 - level
  osum <- summary(object)
  
  # fixed-effects
  x <- osum$coef
  crit.val <- qt(1 - alpha/2, df = x[,3])
  beta.ci <- data.frame(lwr = x[,1] - x[,2] * crit.val,
                        upr = x[,1] + x[,2] * crit.val)
  fix.tab <- cbind(Estimate = x[,1], SE = x[,2], beta.ci, p = x[,5])
  fix.names <- rownames(x)
  
  # random-effects
  varcomp.ci <- confint(object, parm = "theta_")^2
  colnames(varcomp.ci) <- c("lwr", "upr")
  varcor <- as.data.frame(osum$varcor)
  pvals <- ranova(object)$`Pr(>Chisq)`
  rand.tab <- cbind(Estimate = varcor[,4],
                    SE = sqrt(diag(object@vcov_varpar)),
                    varcomp.ci, p = c(pvals[-1], NA))
  rand.names <- varcor[,1]
  
  # r-squared
  Rsq1 <- cor(object@frame[,1], predict(object, re.form = NA))^2
  Rsq2 <- cor(object@frame[,1], predict(object))^2
  Rsq <- c(Rsq1, Rsq2, rep(NA, 3))
  
  # number of observations
  nobs <- c(unname(osum$ngrps), nrow(object@frame), rep(NA, 2))
  
  # combine, round, return
  tab <- rbind(rep(NA, 5), fix.tab, 
               rep(NA, 5), rand.tab, 
               rep(NA, 5), Rsq, nobs)
  rownames(tab) <- c("Fixed effects", fix.names, 
                     "Random effects", rand.names, 
                     "Fit information", "R-squared", "N Obs")
  round(tab, digits)
  
}
```

## 11.2 Table 1: Competence ~ eFACE Total

```
tab1 <- apatab(total.Com)
```

```
## Computing profile confidence intervals ...
```

```
write.csv(tab1, file = "table1.csv")
tab1
```

```
##                 Estimate     SE      lwr    upr     p
## Fixed effects         NA     NA       NA     NA    NA
## (Intercept)       -7.560  0.792   -9.120 -5.999 0.000
## videoAge           0.051  0.010    0.031  0.070 0.000
## videoGenderMale   -0.149  0.338   -0.825  0.527 0.661
## raterAge          -0.045  0.011   -0.066 -0.023 0.000
## raterGenderMale   -0.180  0.389   -0.944  0.583 0.643
## Attractiveness     1.698  0.070    1.562  1.835 0.000
## Total              0.032  0.012    0.008  0.057 0.009
## Random effects        NA     NA       NA     NA    NA
## raterID            9.259  0.043    7.192 11.392 0.000
## videoID            0.495  0.041    0.013  1.026 0.017
## Residual          22.333  0.099   20.609 24.273    NA
## Fit information       NA     NA       NA     NA    NA
## R-squared          0.316  0.623       NA     NA    NA
## N Obs            538.000 61.000 1757.000     NA    NA
```

## 11.3 Table 2: Affiliation ~ eFACE Total

```
tab2 <- apatab(total.Aff)
```

```
## Computing profile confidence intervals ...
```

```
write.csv(tab2, file = "table2.csv")
tab2
```

```
##                 Estimate     SE      lwr    upr     p
## Fixed effects         NA     NA       NA     NA    NA
## (Intercept)       -4.137  0.813   -5.747 -2.526 0.000
## videoAge           0.056  0.013    0.030  0.083 0.000
## videoGenderMale   -1.486  0.450   -2.387 -0.585 0.002
## raterAge          -0.021  0.008   -0.035 -0.006 0.006
## raterGenderMale   -0.259  0.271   -0.792  0.274 0.340
## Attractiveness     1.060  0.055    0.951  1.169 0.000
## Total              0.038  0.016    0.006  0.069 0.021
## Random effects        NA     NA       NA     NA    NA
## raterID            3.122  0.043    2.141  4.153 0.000
## videoID            1.994  0.046    1.135  2.938 0.000
## Residual          14.399  0.079   13.279 15.643    NA
## Fit information       NA     NA       NA     NA    NA
## R-squared          0.253  0.543       NA     NA    NA
## N Obs            533.000 61.000 1718.000     NA    NA
```

## 11.4 Table 3: Dominance ~ eFACE Total

```
tab3 <- apatab(total.Dom)
```

```
## Computing profile confidence intervals ...
```

```
write.csv(tab3, file = "table3.csv")
tab3
```

```
##                 Estimate     SE      lwr    upr     p
## Fixed effects         NA     NA       NA     NA    NA
## (Intercept)       -7.161  0.965   -9.078 -5.244 0.000
## videoAge           0.025  0.017   -0.008  0.059 0.133
## videoGenderMale    1.139  0.567    0.003  2.275 0.049
## raterAge           0.004  0.007   -0.010  0.018 0.565
## raterGenderMale    0.695  0.263    0.179  1.211 0.008
## Attractiveness     0.825  0.056    0.715  0.935 0.000
## Total              0.000  0.020   -0.040  0.040 0.997
## Random effects        NA     NA       NA     NA    NA
## raterID            2.426  0.046    1.464  3.429 0.000
## videoID            3.522  0.054    2.155  5.019 0.000
## Residual          15.108  0.082   13.925 16.425    NA
## Fit information       NA     NA       NA     NA    NA
## R-squared          0.126  0.469       NA     NA    NA
## N Obs            536.000 61.000 1725.000     NA    NA
```

## 11.5 Table 4: Competence ~ eFACE Subscores

```
tab4 <- apatab(sub.Com)
```

```
## Computing profile confidence intervals ...
```

```
write.csv(tab4, file = "table4.csv")
tab4
```

```
##                 Estimate     SE      lwr    upr     p
## Fixed effects         NA     NA       NA     NA    NA
## (Intercept)       -7.027  0.826   -8.655 -5.400 0.000
## videoAge           0.040  0.011    0.018  0.062 0.001
## videoGenderMale   -0.048  0.339   -0.727  0.631 0.888
## raterAge          -0.044  0.011   -0.065 -0.023 0.000
## raterGenderMale   -0.193  0.389   -0.958  0.571 0.620
## Attractiveness     1.686  0.070    1.548  1.823 0.000
## Static            -0.011  0.010   -0.032  0.010 0.309
## Dynamic            0.026  0.008    0.011  0.041 0.001
## Synkinesis         0.003  0.007   -0.010  0.017 0.601
## Random effects        NA     NA       NA     NA    NA
## raterID            9.299  0.043    7.217 11.427 0.000
## videoID            0.428  0.043    0.000  0.870 0.040
## Residual          22.316  0.099   20.598 24.261    NA
## Fit information       NA     NA       NA     NA    NA
## R-squared          0.317  0.623       NA     NA    NA
## N Obs            538.000 61.000 1757.000     NA    NA
```

## 11.6 Table 5: Affiliation ~ eFACE Subscores

```
tab5 <- apatab(sub.Aff)
```

```
## Computing profile confidence intervals ...
```

```
write.csv(tab5, file = "table5.csv")
tab5
```

```
##                 Estimate     SE      lwr    upr     p
## Fixed effects         NA     NA       NA     NA    NA
## (Intercept)       -3.397  0.820   -5.023 -1.772 0.000
## videoAge           0.040  0.014    0.013  0.067 0.005
## videoGenderMale   -1.133  0.418   -1.971 -0.295 0.009
## raterAge          -0.020  0.008   -0.035 -0.006 0.007
## raterGenderMale   -0.273  0.271   -0.805  0.260 0.315
## Attractiveness     1.050  0.055    0.941  1.158 0.000
## Static            -0.020  0.013   -0.046  0.006 0.135
## Dynamic            0.036  0.009    0.017  0.055 0.000
## Synkinesis        -0.010  0.008   -0.027  0.006 0.201
## Random effects        NA     NA       NA     NA    NA
## raterID            3.116  0.043    2.134  4.143 0.000
## videoID            1.512  0.044    0.762  2.181 0.000
## Residual          14.404  0.079   13.285 15.650    NA
## Fit information       NA     NA       NA     NA    NA
## R-squared          0.273  0.542       NA     NA    NA
## N Obs            533.000 61.000 1718.000     NA    NA
```

## 11.7 Table 6: Dominance ~ eFACE Subscores

```
tab6 <- apatab(sub.Dom)
```

```
## Computing profile confidence intervals ...
```

```
write.csv(tab6, file = "table6.csv")
tab6
```

```
##                 Estimate     SE      lwr    upr     p
## Fixed effects         NA     NA       NA     NA    NA
## (Intercept)       -6.780  1.069   -8.907 -4.652 0.000
## videoAge           0.017  0.019   -0.021  0.056 0.375
## videoGenderMale    1.220  0.591    0.037  2.404 0.043
## raterAge           0.004  0.007   -0.010  0.019 0.560
## raterGenderMale    0.693  0.263    0.177  1.210 0.009
## Attractiveness     0.824  0.056    0.713  0.934 0.000
## Static            -0.015  0.018   -0.052  0.021 0.409
## Dynamic            0.008  0.013   -0.018  0.034 0.533
## Synkinesis        -0.003  0.011   -0.026  0.020 0.772
## Random effects        NA     NA       NA     NA    NA
## raterID            2.434  0.046    1.469  3.436 0.000
## videoID            3.605  0.056    2.113  4.931 0.000
## Residual          15.102  0.082   13.921 16.420    NA
## Fit information       NA     NA       NA     NA    NA
## R-squared          0.128  0.469       NA     NA    NA
## N Obs            536.000 61.000 1725.000     NA    NA
```

# 12 Figure 2: eFACE Conditional Effects

Syntax for Figure 2:

```
### initializations
col <- rgb(t(col2rgb("gray75")/256), alpha = 0.5)
par(mfrow = c(3, 4), mar = c(5, 4.5, 2, 2) + 0.1)
yvars <- c("Competence", "Affiliation", "Dominance")
xvars <- c("Static", "Dynamic", "Synkinesis")
xmean <- c(StaticMean, DynamicMean, SynkinesisMean)

### combine model results (total score)
total <- list(total.Com, total.Aff, total.Dom)
total.coef <- list(total.Com.sum$coef, total.Aff.sum$coef, total.Dom.sum$coef)

### combine model results (subscores)
subscore <- list(sub.Com, sub.Aff, sub.Dom)
sub.coef <- list(sub.Com.sum$coef, sub.Aff.sum$coef, sub.Dom.sum$coef)

### loop through the 3 variables
for(j in 1:length(yvars)){
  
  ###### total score results ######
  
  ## get fitted values and coefs
  yhat <- predict(total[[j]])
  coefs <- fixef(total[[j]])
  
  ## find Total effect and df
  indx <- which(names(coefs) == "Total")
  df <- total.coef[[j]][indx,3]
  cval <- qt(0.975, df = df)
  
  ## get residuals w/o Total effect
  rhat <- total[[j]]@frame[,1] - (yhat - total[[j]]@frame[,indx] * coefs[indx])
  
  ## average residuals across raters
  ravg <- tapply(rhat, total[[j]]@frame$videoID, mean)
  xavg <- uniPatients$Total[match(names(ravg), uniPatients$videoID)] - TotalMean
  
  ## sort by Total score (for plotting lines)
  xsort <- sort(xavg, index.return = TRUE)
  xavg <- xavg[xsort$ix]
  ravg <- ravg[xsort$ix]
  
  ## set-up plot (w/ mean added back)
  ylim <- c(-2.25, 2.25)
  plot(xavg + TotalMean, ravg, ylim = ylim,
       xlab = "eFACE Total", ylab = yvars[j],
       cex.lab = 1.5, cex.axis = 1.25, t = "n")
  
  ## confidence interval polygon
  yhat <- xavg * coefs[indx]
  cilo <- yhat - cval * sqrt(total.coef[[j]][indx,2]^2 * xavg^2)
  ciup <- yhat + cval * sqrt(total.coef[[j]][indx,2]^2 * xavg^2)
  polygon(x = c(xavg, rev(xavg)) + TotalMean,
          y = c(cilo, rev(ciup)),
          col = col, border = NA)
  
  ## add points and lines
  points(xavg + TotalMean, ravg)
  abline(h = 0, lty = 3)
  lines(xavg + TotalMean, yhat, lwd = 2, col = "blue")
  
  
  ###### subscore results ######
  
  ## loop through 3 subscores
  for(k in 1:3){
    
    ## get fitted values and coefs
    yhat <- predict(subscore[[j]])
    coefs <- fixef(subscore[[j]])
    
    ## find subscore effect and df
    indx <- which(names(coefs) == xvars[k])
    df <- sub.coef[[j]][indx,3]
    cval <- qt(0.975, df = df)
    
    ## get residuals w/o subscore effect
    rhat <- subscore[[j]]@frame[,1] - (yhat - subscore[[j]]@frame[,indx] * coefs[indx])
    
    ## average residuals across raters
    ravg <- tapply(rhat, subscore[[j]]@frame$videoID, mean)
    xavg <- uniPatients[match(names(ravg), uniPatients$videoID), xvars[k]] - xmean[k]
    
    ## sort by subscore (for plotting lines)
    xsort <- sort(xavg, index.return = TRUE)
    xavg <- xavg[xsort$ix]
    ravg <- ravg[xsort$ix]
    
    ## set-up plot (w/ mean added back)
    plot(xavg + xmean[k], ravg, xlim = c(10, 100), ylim = ylim,
         xlab = paste("eFACE", xvars[k]), ylab = yvars[j],
         cex.lab = 1.5, cex.axis = 1.25, t = "n")
    
    ## confidence interval polygon
    yhat <- xavg * coefs[indx]
    cilo <- yhat - cval * sqrt(sub.coef[[j]][indx,2]^2 * xavg^2)
    ciup <- yhat + cval * sqrt(sub.coef[[j]][indx,2]^2 * xavg^2)
    polygon(x = c(xavg, rev(xavg)) + xmean[k],
            y = c(cilo, rev(ciup)),
            col = col, border = NA)
    
    ## add points and lines
    points(xavg + xmean[k], ravg)
    abline(h = 0, lty = 3)
    lines(xavg + xmean[k], yhat, lwd = 2, col = "blue")
    
  }
  
}
```

```
dev.copy2pdf(file = "fig2.pdf")
```

```
## quartz_off_screen 
##                 2
```

# 13 Power Analyses

## 13.1 lmer.power Function

First, we will create a function for estimating the power of
fixed-effects in LMER models. Our function leverages the built-in
functionality of the *simulate* function in the
**lme4** package, which can be used to simulate data new
response data from a fit LMER model.

```
lmer.power <-
  function(object, nsim = 1000, re.form = NA,
           alpha = 0.05, verbose = TRUE){
    # post-hoc power for lmer fixed effects

    # initial checks
    if(class(object) != "lmerModLmerTest") {
      stop("Input 'object' must be of class 'lmerModLmerTest'.")
    }
    nsim <- as.integer(nsim[1])
    if(nsim < 1L) stop("Input 'nsim' must be a postive integer.")

    # get original formula and data
    theform <- formula(object)
    thedata <- object@frame

    # print progress bar?
    if(verbose) pbar <- txtProgressBar(max = nsim)

    # initialize vector for results
    betas <- fixef(object)
    nterms <- length(betas)
    power <- rep(0, nterms)
    names(power) <- names(betas)

    # loop through nsim
    for(i in 1:nsim){

      if(verbose) setTxtProgressBar(pbar, i)

      # simulate new data
      thedata[,1] <- simulate(object, re.form = re.form)

      # refit lmer model with new data (using lmerTest)
      newmod <- lmerTest::lmer(theform, data = thedata)

      # test significance of term
      newsum <- summary(newmod)
      power <- power + (newsum$coefficients[,5] < alpha)

    } # end for(i in 1:nsim)

    if(verbose) close(pbar)

    # return results
    res <- list(power = power/nsim, alpha = alpha,
                nsim = nsim, re.form = re.form)
    return(res)

  }
```

## 13.2 eFACE Total Score Power

### 13.2.1 Competence

```
set.seed(1)
total.Com.pwr <- lmer.power(total.Com, verbose = FALSE)
```

### 13.2.2 Affiliation

```
set.seed(1)
total.Aff.pwr <- lmer.power(total.Aff, verbose = FALSE)
```

### 13.2.3 Dominance

```
set.seed(1)
total.Dom.pwr <- lmer.power(total.Dom, verbose = FALSE)
```

### 13.2.4 Table Results

```
total.pwr.tab <- data.frame(Com = total.Com.pwr$power,
                            Aff = total.Aff.pwr$power,
                            Dom = total.Dom.pwr$power)
write.csv(total.pwr.tab, file = "power-total.csv")
total.pwr.tab
```

```
##                   Com   Aff   Dom
## (Intercept)     1.000 0.998 1.000
## videoAge        0.999 0.988 0.324
## videoGenderMale 0.066 0.900 0.532
## raterAge        0.981 0.767 0.071
## raterGenderMale 0.091 0.165 0.748
## Attractiveness  1.000 1.000 1.000
## Total           0.727 0.642 0.058
```

## 13.3 eFACE Subscore Power

### 13.3.1 Competence

```
set.seed(1)
sub.Com.pwr <- lmer.power(sub.Com, verbose = FALSE)
```

### 13.3.2 Affiliation

```
set.seed(1)
sub.Aff.pwr <- lmer.power(sub.Aff, verbose = FALSE)
```

### 13.3.3 Dominance

```
set.seed(1)
sub.Dom.pwr <- lmer.power(sub.Dom, verbose = FALSE)
```

### 13.3.4 Table Results

```
sub.pwr.tab <- data.frame(Com = sub.Com.pwr$power,
                          Aff = sub.Aff.pwr$power,
                          Dom = sub.Dom.pwr$power)
write.csv(sub.pwr.tab, file = "power-subscore.csv")
sub.pwr.tab
```

```
##                   Com   Aff   Dom
## (Intercept)     1.000 0.988 1.000
## videoAge        0.937 0.847 0.141
## videoGenderMale 0.047 0.767 0.555
## raterAge        0.980 0.758 0.071
## raterGenderMale 0.097 0.177 0.743
## Attractiveness  1.000 1.000 1.000
## Static          0.178 0.315 0.136
## Dynamic         0.911 0.970 0.108
## Synkinesis      0.093 0.250 0.078
```
